# Risk of domperidone induced severe ventricular arrhythmia

**DOI:** 10.1038/s41598-020-69053-4

**Published:** 2020-07-22

**Authors:** Byeong Geun Song, Yeong Chan Lee, Yang Won Min, Kyunga Kim, Hyuk Lee, Hee Jung Son, Poong-Lyul Rhee

**Affiliations:** 1 Department of Medicine, Samsung Medical Center, Sungkyunkwan University School of Medicine, 81 Irwon-ro, Gangnam-gu, Seoul, 06351 South Korea; 2 Department of Digital Health, Samsung Advanced Institute for Health Sciences and Technology (SAIHST), Sungkyunkwan University, Seoul, South Korea; 3 Statistics and Data Center, Research Institute for Future Medicine, Samsung Medical Center, Seoul, South Korea

**Keywords:** Gastrointestinal system, Drug safety, Pharmacology

## Abstract

There has been controversy over the cardiovascular safety of domperidone, attributable to the lack of a well-designed study as well as inconsistent results. This study aimed to examine the risk of severe domperidone-induced ventricular arrhythmia (VA), compared to mosapride, itopride, or non-use of all three prokinetics, in the general population. We conducted a population-based, self-controlled case series analysis. Enrolled subjects were individuals who were diagnosed with severe VA and were prescribed domperidone, mosapride, or itopride from 2003 to 2013 in the National Health Insurance Service-National Sample Cohort. The incidence rate ratio for severe VA was measured during exposure to prokinetics and compared with unexposed periods and itopride (no-proarrhythmic effect)-exposure periods, as control. A total of 2,817 subjects were included. Domperidone, mosapride, or itopride use was associated with increased risk of severe VA, compared with non-use (adjusted incidence rate ratios (IRR) of 1.342 (95% CI 1.096–1.642), 1.350 (95% CI 1.105–1.650), and 1.486 (95% CI 1.196–1.845), respectively). The risk of severe domperidone-induced VA was lower, compared to that of itopride [adjusted IRR of 0.548 (95% CI 0.345–0.870)]. Of the subjects who had been prescribed all three prokinetics, domperidone-exposure was associated with a lower risk of severe VA, compared to itopride-exposure (crude IRR, 0.571; 0.358–0.912). Mosapride-exposure did not show IRR difference for severe VA, compared to itopride-exposure. Domperidone, mosapride, or itopride use is associated with an increased risk of severe VA. However, the magnitude of association was modest and domperidone use does not increase further the risk, compared with other prokinetics.

## Introduction

Domperidone is effective for treating nausea and vomiting, delayed gastric emptying, and dyspepsia^1,2^ and is preferred to other commonly prescribed prokinetic agents such as metoclopramide and mosapride^2^. It is not associated with central nervous system side effects because it does not readily cross the blood–brain barrier^1,2^. Domperidone is more effective than other prokinetic agents in treating gastroparesis^3,4^. and exhibits concomitant central antiemetic activity through dopamine receptors within the chemoreceptor trigger zone^5^. Despite all these advantages, the clinical use of oral domperidone is currently limited to individuals with an FDA Investigational New Drug exemption (treatment-refractory gastroparesis), owing to the risk of severe ventricular arrhythmia (VA)^2^.

Cardiac safety concerns were raised in the 1980s, when several serious cardiac events were reported^6–9^. Intravenous domperidone has since been withdrawn from the market, with limited use of oral domperidone because of a possible association with cardiotoxicity^10–14^. Although case series and retrospective case–control studies exist on the association between domperidone use and severe VA, there has been no prospective study or randomized controlled study with an appropriate control. Thus, we aimed to investigate the cardiac safety issue of domperidone by conducting a self-controlled case series (SCCS) using a large population-based cohort and an appropriate control.

## Results

**Figure 1:**
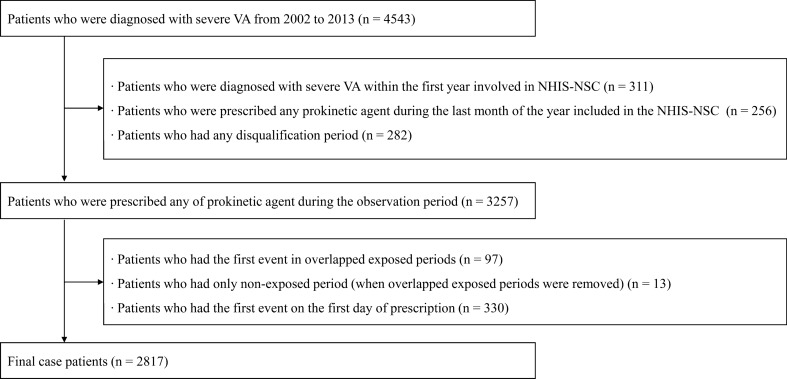
Flowchart for the selection of the study population.

We identified 2,817 patients with at least one incident of severe VA and exposure to domperidone, mosapride, or itopride between 2003 and 2013 (Fig. 1). The study medication at cohort entry was domperidone in 70.3%, mosapride in 70.9%, and itopride in 60.8% of the patients (Table 1). The median age at cohort entry was 47 years (45–49 years) and 46.0% of patients were males. Severe VA was observed in 2,487 cases during 25,130.7 person-year non-exposure periods (incidence rate, 9.9 cases per 100 person-years), 117 cases during 813.95 person-year domperidone-exposure periods (incidence rate, 14.4 cases per 100 person-years), 114 cases during 776.6 person-year mosapride-exposure periods (incidence rate 14.7 cases per 100 person-years), and 99 cases during 608.5 person-year itopride-exposure periods (incidence rate, 16.3 cases per 100 person-years) (Table 2). Severe VA crude IRRs for domperidone, mosapride, and itopride, compared to non-exposure were as follows: 1.345 (95% CI 1.100–1.646, *p* = 0.004), 1.541 (95% CI 1.266–1.877, *p* < 0.001), and 1.635 (95% CI 1.319–2.026, *p* < 0.001) (Table 3). When adjusted for confounders (age, sex, and co-morbidities), the association remained similar.

**Table 1:** Characteristics of prokinetic users during the study period (*n* = 2,817).

Variables	*N* (%)
**Age groups**	
0–14	149 (5.3)
15–29	316 (11.2)
30–44	669 (23.7)
45–59	872 (31.0)
60–74	687 (24.4)
75 +	124 (4.4)
**Sex**	
Male	1,295 (46.0)
**Underlying disease**	
Structural heart disease	594 (21.1)
Hypertension	942 (33.4)
Diabetes mellitus	569 (20.2)
Dyslipidemia	733 (26.0)
Arrhythmia	125 (4.4)
**Number of prokinetics users**	
Domperidone	1979 (70.3)
Mosapride	1996 (70.9)
Itopride	1714 (60.8)

The data are presented as numbers (percentages).

**Table 2:** Incidence rate of severe VA.

	Non-exposure	Domperidone-exposure	Mosapride-exposure	Itopride-exposure
Number of patients	2,817	1979	1996	1714
Total risk periods (person-year)	25,130.7	813.95	776.6	608.5
Severe VA events per risk period	2,487	117	114	99
Incidence rate per 100 person-years	9.9	14.4	14.7	16.3

*VA* ventricular arrhythmia.

**Table 3:** Incident rate ratio of severe VA associated with prokinetic use compared with non-exposure.

	N	IRR (95% CI)		
		Unadjusted model	Adjusted model 1	Adjusted model 2
Domperidone-exposure	1979	1.345 (1.100–1.646)	1.351 (1.104–1.653)	1.342 (1.096–1.642)
Mosapride-exposure	1996	1.541 (1.266–1.877)	1.491 (1.224–1.816)	1.350 (1.105–1.650)
Itopride-exposure	1714	1.635 (1.319–2.026)	1.596 (1.288–1.979)	1.486 (1.196–1.845)

Model 1: adjusted for age and sex.

Model 2: adjusted for age, sex, and co-morbidities.

*VA* ventricular arrhythmia, *IRR* incident rate ratio, *CI* confidence interval.

Of the 918 patients who had been prescribed all three prokinetics, severe VA was observed in 765 cases during the 8,332 person-year unexposed periods, 36 cases during the 397.73 person-year domperidone-exposure periods, 57 cases during the 384.1 person-year mosapride-exposure periods, and 60 cases during the 354.4 person-year itopride-exposure periods. Domperidone-exposure was not associated with increased risk of severe VA (crude IRR; 0.948, 0.672–1.338, *p* = 0.762), whereas mosapride and itopride-exposure were associated with increased risk of severe VA, compared to non-exposure. After adjusting for confounders (age, sex, and co-morbidities), these associations remained unchanged (Table 4).

**Table 4:** Incident rate ratio of severe VA associated with prokinetic use compared with non-exposure among the patients who had been exposed to all three prokinetics.

*N* = 918	IRR (95% CI)		
	Unadjusted model	Adjusted model 1	Adjusted model 2
Domperidone-exposure	0.948 (0.672–1.338)	0.951 (0.674–1.342)	0.958 (0.679–1.353)
Mosapride-exposure	1.630 (1.234–2.154)	1.583 (1.197–2.093)	1.472 (1.118–1.966)
Itopride-exposure	1.874 (1.425–2.466)	1.842 (1.400–2.424)	1.783 (1.353–2.348)

Model 1: Adjusted for age.

Model 2: Adjusted for age and co-morbidities.

*VA* ventricular arrhythmia, *IRR* incident rate ratio, *CI* confidence interval.

Of the 2,817 patients who had been prescribed any of three prokinetics, severe VA was observed in 2,487 cases during the 25,130.7 person-year unexposed periods, 117 cases during the 814 person-year domperidone-exposure periods, 114 cases during the 776.6 person-year mosapride-exposure periods, and 99 cases during the 608.5 person-year itopride-exposure periods (Table 5). All prokinetic-exposure was associated with increased risk of severe VA, compared to non-exposure.

**Table 5:** Incident rate ratio of severe VA associated with prokinetic use compared with non-exposure among the patients who had been exposed to any prokinetic.

*N* = 2,817	IRR (95% CI)		
	Unadjusted model	Adjusted model 1	Adjusted model 2
Domperidone-exposure	1.341 (1.097–1.638)	1.345 (1.101–1.644)	1.336 (1.093–1.634)
Mosapride-exposure	1.554 (1.277–1.891)	1.500 (1.232–1.826)	1.324 (1.084–1.616)
Itopride-exposure	1.661 (1.341–2.056)	1.621 (1.309–2.007)	1.501 (1.210–1.862)

Model 1: adjusted for age.

Model 2: adjusted for age and co-morbidities.

*VA* ventricular arrhythmia, *IRR* incident rate ratio, *CI* confidence interval.

The IRRs of severe domperidone- and mosapride-induced VA were compared to itopride-exposure. Domperidone-exposure was associated with a lower risk of severe VA, compared to itopride-exposure (crude IRR, 0.512; 0.328–0.801; *p* = 0.003), whereas mosapride-exposure failed to show any significant IRR difference for severe VA, compared to itopride-exposure (crude IRR, 1.111; 0.772–1.598; *p* = 0.571) (Table 6). After adjusting for confounders (age and co-morbidities), these associations remained unchanged. Also, of the patients who had been prescribed all three prokinetics, domperidone-exposure was associated with a lower risk of severe VA, compared to itopride-exposure (crude IRR, 0.571; 0.358–0.912), whereas mosapride-exposure did not show any significant IRR difference for severe VA, compared to itopride-exposure (crude IRR, 0.985; 0.655–1.481) (Table 7). Similarly, of the patients who had been exposed to any prokinetics, domperidone-exposure was associated with a lower risk of severe VA, compared to itopride-exposure (IRR, 0.678; 0.462–0.997), whereas mosapride-exposure did not show significant IRR difference for severe VA, compared to itopride-exposure (crude IRR 0.950; 0.672–1.343) (Table 8).

**Table 6:** Incident rate ratio of severe VA associated with prokinetic use compared with itopride as the control.

	N	IRR (95% CI)		
		Unadjusted model	Adjusted model 1	Adjusted model 2
Domperidone-exposure	1,165	0.512 (0.328–0.801)	0.504 (0.321–0.792)	0.548 (0.345–0.870)
Mosapride-exposure	1,262	1.111 (0.772–1.598)	1.147 (0.793–1.657)	1.132 (0.778–1.648)

Model 1: adjusted for age.

Model 2: adjusted for age and co-morbidities.

*VA* ventricular arrhythmia, *IRR* incident rate ratio, *CI* confidence interval.

**Table 7:** Incident rate ratio of severe VA associated with prokinetic use compared with itopride as the control among the patients who had been exposed to all three prokinetics.

*N* = 918	IRR (95% CI)		
	Unadjusted model	Adjusted model 1	Adjusted model 2
Domperidone-exposure	0.571 (0.358–0.912)	0.551 (0.344–0.883)	0.573 (0.354–0.925)
Mosapride-exposure	0.985 (0.655–1.481)	1.014 (0.672–1.529)	0.973 (0.641–1.477)

Model 1: adjusted for age.

Model 2: adjusted for age and co-morbidities.

*VA* ventricular arrhythmia, *IRR* incident rate ratio, *CI* confidence interval.

**Table 8:** Incidence rate ratio of severe VA associated with prokinetic use compared with itopride as the control among the patients who had been exposed to any prokinetic.

*N* = 2,817	IRR (95% CI)		
	Unadjusted model	Adjusted model 1	Adjusted model 2
Domperidone-exposure	0.678 (0.462–0.997)	0.661 (0.449–0.973)	0.746 (0.503–1.106)
Mosapride-exposure	0.950 (0.672–1.343)	0.967 (0.683–1.371)	0.926 (0.650–1.321)

Model 1: adjusted for age.

Model 2: adjusted for age and co-morbidities.

*VA* ventricular arrhythmia, *IRR* incident rate ratio, *CI* confidence interval.

## Discussion

Initially, domperidone was commonly used to treat nausea, vomiting, and gastroparesis, owing to its effectiveness^2^. However, of late, given that several case reports and retrospective studies have reported an association between domperidone and severe VA, its use has been strictly restricted. Although some meta-analyses indicate that domperidone may not be associated with the risk of overall cardiovascular (CV) events and QT prolongation^15–17^, many clinicians hesitate to prescribe it to their patients. Thus, it is essential to conduct well-designed studies to clarify the association between domperidone and severe VA. We conducted a self-controlled case series based on a large population cohort, which could enable the adjustment of all fixed time-independent confounders during the observation period.

Our study suggested that domperidone may be associated with increased risk of severe VA. However, considering the weak association (adjusted IRR, 1.342), it is less likely to have a causal relationship^18^. This modest association, referred to as the “zone of bias,” can be easily susceptible to undetected biases, particularly residual confounding^18,19^. Therefore, the causal inference from observational studies must be carefully interpreted. Tables 6, 7 and 8 show that domperidone is less likely to be associated with severe VA, compared with itopride, known to be the safest prokinetic agent^20^. Thus, it can be inferred that the weak association between domperidone and severe VA could be largely attributed to undetected bias.

Published data on potential domperidone-induced adverse CV events are largely limited to case reports and case–control studies^15,21–23^. Given the nature of the retrospective study design, it is inherently impossible to avoid bias. We used the self-controlled case series design to effectively avoid confounding and bias, unlike the traditional case–control or cohort designs. In this study design, comparisons were made entirely intra-personally^24^. Therefore, it is self-controlled and accounts for any factors or characteristics that remain constant over the observation period^24^. Our study results are consistent with a recent meta-analysis on the cardiac safety profile of domperidone^15^. Serhat et al. conducted the meta-analysis using nine studies and reported that low-dose domperidone may not be associated with the overall risk of CV events and does not result in QT prolongation^15^.

Domperidone displays an affinity for the hERG/Kv11.1 channel and has been linked to drug induced long QT syndrome, torsades de pointes (TdP), and sudden cardiac death (SCD)^5,25,26^. However, an exaggerated QTc response, TdP, and/or SCD are rarely observed after its oral administration^5^. This phenomenon may be attributable to the vastly lower intra-cardiomyocyte concentrations of these medications, resulting from the combined effects of hepatic drug metabolism and intracellular drug accumulation on the bioavailability^27–29^. Practically, severe domperidone-induced VA rarely occurs, considering the frequency of domperidone prescription. Severe VA is likely to occur when multifactorial occurrences such as drug-drug interactions (co-administration of potent CYP3A4 inhibitors), patient-specific risk factors, and underlying genetic predisposition, are combined^5^. Thus, if domperidone is prescribed after careful consideration of drug interactions and underlying disease, the risk of severe VA would be minimal.

An important advantage of this study is the use of the self-controlled case series design, which cancels out time-invariant factors. Furthermore, we analyzed the incidence rate ratio of severe VA for other commonly prescribed prokinetics; itopride and mosapride. We equally compared domperidone-induced severe VA risk to that of itopride, for the first time. When prokinetics were prescribed for early symptoms of undiagnosed heart disease, it was possible to exaggerate the association between prokinetics and severe VA. However, we could effectively adjust this potential bias by comparing other prokinetics. Although only a few studies have used case–control design with incomplete controls, there have been no studies using a robust study design with appropriate controls or prospective studies. We also used a large nationwide cohort representing the general population. We followed-up 2,817 severe VA patients extracted from over 1 million subjects for up to 11 years.

As a limitation, the current study failed to investigate the association between prokinetic dose and severe VA. Secondly, it was unfeasible to take concomitant medication related to QT prolongation into consideration. However, given that we adopted a self-controlled case series design while using other prokinetics as control, it was possible to adjust for confounding factors.

Our study is indicative of insufficient evidence to support any association between the use of domperidone and increased severe VA risk, compared to other prokinetics. Thus, domperidone can be safely prescribed to patients in need of prokinetics.

## Methods

### Data source

Data was obtained from the National Health Insurance Service National Sample Cohort (NHIS-NSC) in South Korea^30^. The NHIS-NSC is a population-based retrospective sample cohort and consists of 1,025,340 individuals who were selected as representatives of the South Korean population, by stratified random sampling from 46,605,433 individuals who were eligible for the National Health Insurance Service in 2002. The cohort contains the de-identified information on personal information, disease diagnosis, drug prescription, medical treatment and death status from January 1st, 2002 till December 31st, 2013. Infants were included annually into the cohort as replacements for individuals who had been excluded due to death or disqualification from the national health insurance owing to situations such as emigration. The study was performed in accordance with the ethical guidelines of the Declaration of Helsinki. The protocol of this study was approved by the Institutional Review Board (No. 2018-11-047) at Samsung Medical Center. Since this study was based on retrospective analysis of existing data, the requirement of obtaining informed consent was waived by the Institutional Review Board.

### Definition

International Classification of Diseases, Tenth Revision (ICD-10) code were used to define severe VA. Patients who were diagnosed with I47.2 (ventricular tachycardia), I49.0 (ventricular fibrillation and flutter), and I49.8 (other specified cardiac arrhythmias) were classified as having severe VA.

### Study population

Figure 1 shows a flow chart for the selection of the study population. Eligible subjects were individuals with at least one severe VA diagnosis in the NHIS-NSC. To ensure that all study subjects had at least one severe VA-free year, we eliminated subjects who were diagnosed with severe VA within the same year of inclusion into the NHIS-NSC. Furthermore, we excluded subjects with prescribed prokinetic agents such as domperidone, itopride, and mosapride during the last month of the year of inclusion into the NHIS-NSC, to ensure at least one prokinetic agent-free month. Subjects were further excluded if they had any disqualification during the course of the observations.

Selected subjects were then prescribed any prokinetic agent during the observation period. Exposed periods and unexposed periods were identified for each agent. After eliminating overlapping exposure periods, we further excluded subjects whose observation period no longer contained the 1st incident severe VA diagnosis or any exposed period. Subjects were equally eliminated if the 1st incident diagnosis occurred within the first 2 days of exposure, to ensure corresponding incidence.

### Study design

The SCCS method is a case-only design in which a cohort consists only of cases (individuals with the outcome or adverse event of interest)^31^. The aim of the SCCS method was to investigate the association between the outcome and exposure of interest, by comparing the incidence rate during risk-increased periods due to exposure (exposed periods), with the incidence rate at other times (unexposed periods), intra-personally^24^. The advantages of this method are two-folds. Firstly, the possible confounding effects of any time-invariant covariates are automatically prevented. Secondly, no separate control selection is required. It can be beneficial in situations where finding suitable controls is challenging.

The outcome of interest was the incidence of severe VA. For individuals with multiple incidences, only the 1st incident diagnosis was included in analyses because the exposure pattern could be modified after incidence occurrence. However, the information on multiple incidences was used to define outcome periods and observation periods.

For individuals with a single incidence, the outcome period was defined as 1 month post-incidence (30 days from the date of the incident diagnosis). For those with multiple incidences, the outcome period for the 1st incidence was defined as post 1-month after the 1st incident diagnosis. If this period overlapped with the post 1-month period(s) after the other incident diagnoses, the outcome period was extended by combining all overlapped periods. The last follow-up date was defined as the earliest date between December 31st, 2013 and the date of death, disqualification, or the 2nd incident diagnosis, as relevant. We defined the observation period as the time from the first date in the year after inclusion into the cohort until the last follow-up date, excluding the outcome period. The observation period was further partitioned into the exposed and unexposed periods.

**Figure 2:**
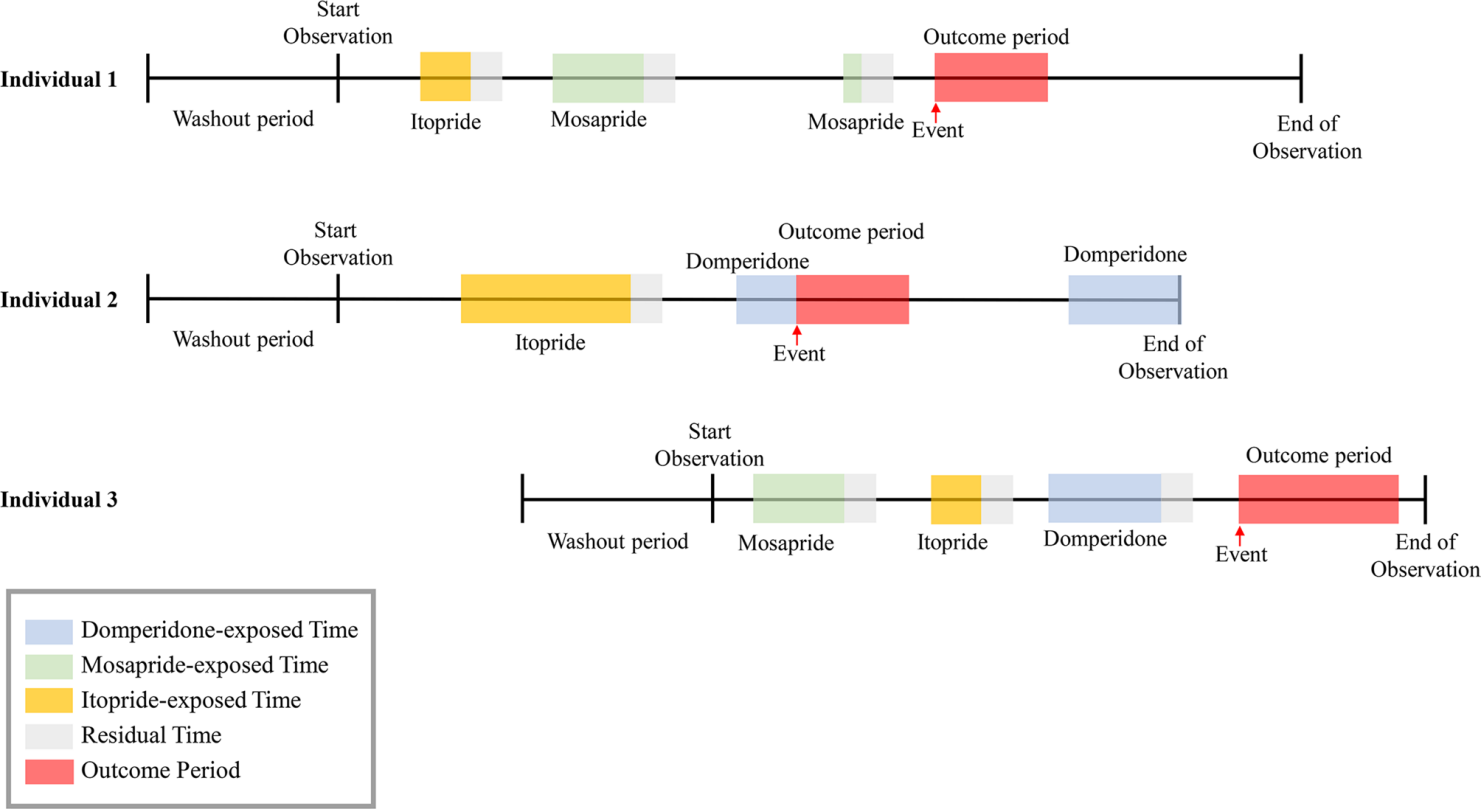
Pictorial representation of the study design. This figure illustrates three individuals prescribed prokinetics during their observation period. All subjects included in the analyses had an incident of severe ventricular arrhythmia (VA) and at least one prescription for domperidone, mosapride, or itopride. The observation period consists of the exposed and unexposed period. The colored exposed period indicates the agent-exposed time (blue, green, or yellow) or residual time (gray). The uncolored time interval between the exposed periods represents the unexposed period. Severe VA can occur during the exposed period (agent-exposed time and the residual time) or the unexposed period. The date of the incident diagnosis indicates the starting point of the outcome period (red). The last follow-up date was defined as the time between the earliest date (including December 31st, 2013) and the date of death, disqualification, or the 2nd incident diagnosis, as relevant.

**Figure 3:**
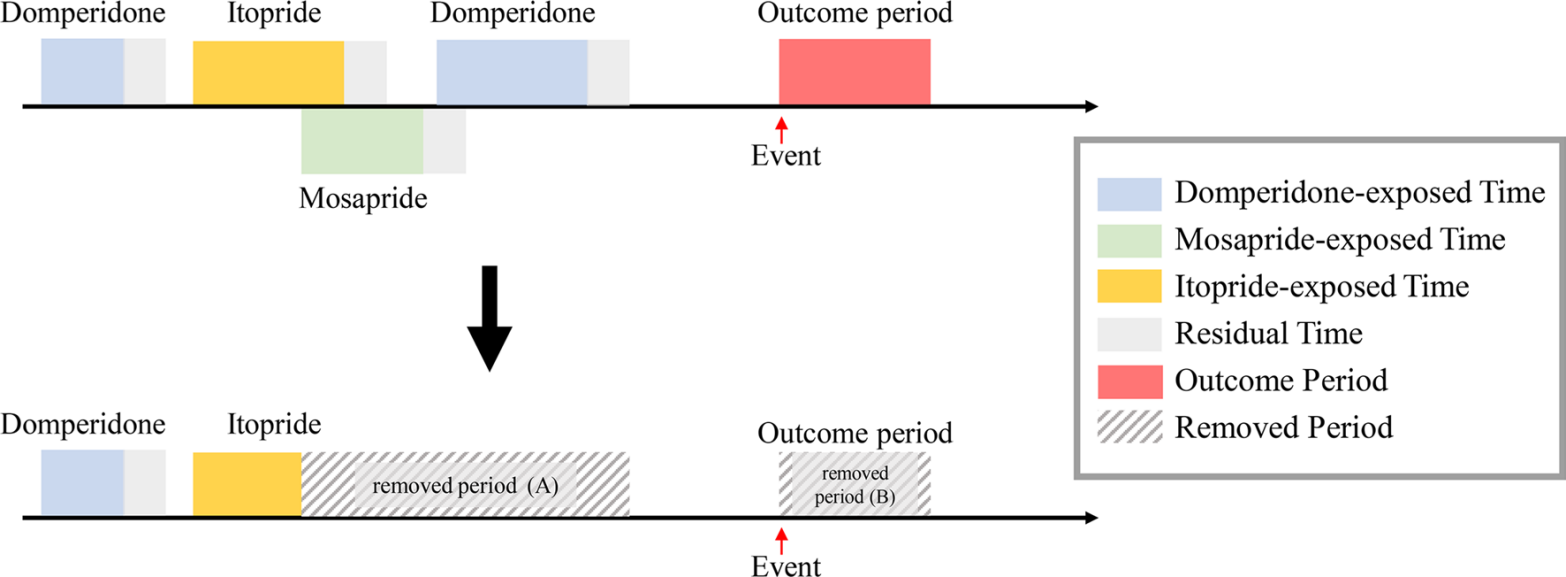
An example of periods eliminated from analyses: (**a)** partial observation period with overlapped exposed times and (**b)** whole outcome period. To avoid possible confounders, such as residual effect created by other agents and the outcome event, sections of the exposed and outcome periods were eliminated from the analyses. Where exposed periods for different agents overlapped, the 1st period was reduced by defining its last date as the first date of the 2nd period.

The exposed period consisted of two parts: the agent-exposed time and the residual time (Fig. 2). We defined the agent-exposed time as the total consecutive days in single or multiple prescriptions for each agent. Taking possible residual efficacy into account, the 30 days following agent-exposed time were considered as the residual time. If exposed periods for different agents overlapped, the first period was shortened by defining its last date as the first date of the 2nd period, and the following periods removed as unobserved (Fig. 3), to avoid confounding effects from different agents. All time intervals other than the exposed periods within the whole observation period were defined as unexposed periods.

## Statistical analyses

The conditional Poisson regression was used to estimate the incidence rate ratios at 95% confidence intervals (CI), which indicated the relative risks of exposure to prokinetic agents. While controlling the time-invariant covariates by the SCCS design, we further adjusted for time-dependent covariates: age group (< 50 versus ≥ 50) and the history of severe VA-related diseases such as structural heart disease, hypertension, diabetes mellitus, and dyslipidemia.

Data preparation and statistical analyses were conducted with R software (version 3.5.0, Vienna, Austria)^32^ and its packages, including gnm^33^. A *P* value < 0.05 was considered statistically significant.

## Abbreviations

VA: Ventricular arrhythmia
SCCS: Self-controlled case series
NHIS-NSC: National Health Insurance Service National Sample Cohort
CI: Confidence intervals
CV: Cardiovascular
TdP: Torsades de pointes
SCD: Sudden cardiac death
